# Potential Efficacy of Luteolin in Cutaneous Squamous Cell Carcinoma: A Combined In Vitro and In Vivo Study

**DOI:** 10.3390/biom16050737

**Published:** 2026-05-18

**Authors:** Yuyang Guo, Xin Wang, Yuan Gao, Yan Xu, Zesen Fang, Silin Liu, Haonan Dong, Jianghan Luo, Lijun Yan

**Affiliations:** School of Pharmacy, Engineering Research Center of Natural Antineoplastic Drugs, Ministry of Education, Harbin University of Commerce, Harbin 150076, China; guoyuyangy023@s.hrbcu.edu.cn (Y.G.); wang3au@163.com (X.W.); xuyany023@s.hrbcu.edu.cn (Y.X.); fangzeseny024@s.hrbcu.edu.cn (Z.F.); liusilin@s.hrbcu.edu.cn (S.L.); donghaonany025@s.hrbcu.edu.cn (H.D.); 102690@hrbcu.edu.cn (J.L.); ylj@hrbcu.edu.cn (L.Y.)

**Keywords:** luteolin, cSCC, apoptosis, A431, EGFR/PI3K/AKT

## Abstract

Cutaneous squamous cell carcinoma (cSCC) is a common malignant skin tumor with invasive potential and risk of recurrence. This study investigated the anti-cSCC effects of luteolin in vitro and in vivo and explored the associated molecular mechanisms. The effects of luteolin on A431 cell viability were assessed by CCK-8 assay, and apoptosis was analyzed by Annexin V-FITC/propidium iodide (PI) double staining. qRT-PCR and Western blot analyses were performed to evaluate apoptosis-related factors and the EGFR/PI3K/AKT signaling pathway. Molecular docking was further conducted to explore the potential interactions of luteolin with EGFR/PI3K/AKT signaling-related proteins and apoptosis-associated proteins. In vivo, a two-stage skin carcinogenesis model induced by 7,12-dimethylbenz[a]anthracene (DMBA) and croton oil was used to evaluate the antitumor activity of luteolin. Luteolin significantly inhibited A431 cell viability and promoted apoptosis in a concentration-dependent manner. Moreover, luteolin increased Bax expression and decreased Bcl-2 expression at both the mRNA and protein levels. Mechanistically, luteolin suppressed the phosphorylation of EGFR, PI3K, and AKT. Molecular docking suggested that luteolin could interact with EGFR, PIK3CA, AKT, Bax, and Bcl-2, providing supportive in silico evidence for its potential modulation of EGFR/PI3K/AKT signaling and apoptosis-related proteins. In vivo, luteolin alleviated body weight loss, achieved a tumor nodule inhibition rate of 45.28%, significantly improved spleen and thymus indices (*p* < 0.05), and ameliorated histopathological damage in skin tissues. In addition, immunohistochemical analysis showed that luteolin reduced Ki-67 expression. These results indicate that luteolin exerts anti-cSCC effects in vitro and in vivo, possibly through modulation of the EGFR/PI3K/AKT signaling pathway and apoptosis-related proteins.

## 1. Introduction

Cutaneous squamous cell carcinoma (cSCC) is a non-melanoma skin cancer (NMSC) characterized by keratinocyte malignancy, accounting for approximately 20% of all skin cancers [1,2]. According to statistics, the total number of skin cancer cases worldwide in 2019 was as high as 6.35 million [3]. As reported in WHO’s 2020 global cancer statistics, the number of new NMSC cases globally reached around 1.2 million (excluding basal cell carcinoma) worldwide, accounting for 6.2% of the total new cancer cases [4]. The pathogenesis of cSCC is a complex, multistep process orchestrated by the dysregulation of multiple molecular pathways and cellular processes, with UV radiation as the primary etiological factor. cSCC has become a common disease that poses a major threat to human health and life. Additionally, surgical resection remains the primary therapeutic modality for localized cSCC currently; however, due to inherent risks, including tumor invasiveness and incomplete excision, a substantial recurrence potential persists, especially in high-risk lesions [5]. In recent years, the incidence of cSCC has continued to rise, driven by population aging, increased cumulative ultraviolet radiation exposure, and the expanding high-risk population, including organ transplant recipients and individuals under chronic immunosuppression [6]. The search for highly effective and safe natural medicines has practical implications for preventing disease or slowing or reversing its progression.

At the molecular level, the pathogenesis of cSCC is driven by aberrant activation of multiple oncogenic signaling cascades. Among them, the dysregulated epidermal growth factor receptor (EGFR) activation, commonly observed in SCC tumors, triggers downstream phosphatidylinositol 3-kinase/protein kinase B (PI3K/AKT) signaling pathway activation, and promotes enhanced cell cycle progression, angiogenesis, and resistance to apoptosis [7]. In recent years, EGFR inhibitors have been effective therapeutic targets for cSCC. For example, EGFR inhibitors like cetuximab have shown clinical efficacy in head and neck squamous cell carcinoma (HNSCC) [8]. Meanwhile, AKT signaling exhibits stage-dependent dynamic changes during cSCC progression: its activity is reduced in premalignant lesions but broadly elevated in invasive cSCC, suggesting that the AKT pathway is involved in malignant transformation and also represents a potential therapeutic vulnerability [9,10]. In addition, review studies on the progression from actinic keratosis to invasive cSCC have indicated that signaling pathways including PI3K/AKT/mTOR, EGFR, and MAPK serve as critical molecular foundations for early lesion progression and therapeutic intervention [11]. At present, the treatment of advanced cutaneous squamous cell carcinoma (cSCC) has achieved remarkable advances owing to the application of PD-1 immune checkpoint inhibitors. Nevertheless, its clinical utility is still limited by immune-related adverse events, primary non-response in certain patients, and acquired drug resistance [12,13]. Therefore, it is of great research significance to explore novel agents with favorable safety profiles that can effectively modulate the EGFR/PI3K/AKT signaling network.

**Figure 1:**
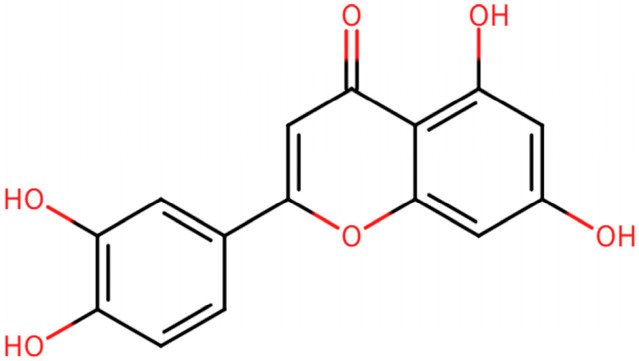
Chemical structure of luteolin.

Luteolin is widely found in a variety of food and medicinal plants and belongs to the flavonoid subclass (Figure 1). Existing as yellow needle-shaped crystals with satisfactory stability under ambient temperature and pressure, this flavonoid has garnered widespread attention in oncology and pharmacology investigations owing to its versatile and potent bioactivities, including anti-inflammatory, antioxidant, and antitumor effects, and has shown a favorable safety profile in preclinical studies [14]. Luteolin exhibits antitumor activities in numerous cancers, such as breast cancer [15,16], liver cancer, lung cancer [17,18], and colon cancer [19]. The anticancer properties of luteolin are mainly related to inhibition of tumor cell proliferation, restraint of neoplasm invasion and metastasis, induction of apoptotic cell death, blockage of angiogenesis, and modulation of cell signaling pathways [20,21,22]. Emerging evidence indicates that luteolin can directly inhibit EGFR tyrosine kinase activity in pancreatic cancer cells and suppress AKT phosphorylation and mTOR signaling in glioblastoma models [23,24]. Luteolin is also able to restrain the expression of p-EGFR, p-STAT3, and p-AKT induced by EGFR in MCF-7 cells, thereby exerting inhibitory activity against breast cancer cells [25]. Additionally, in models such as esophageal squamous cell carcinoma, luteolin can also enhance chemosensitivity by downregulating the FAK/PI3K/AKT signaling pathway, while exhibiting low toxicity in in vivo experiments [26]. These findings suggest that luteolin may exert its antitumor effects by targeting the EGFR/PI3K/AKT signaling axis.

Nevertheless, although the PI3K/AKT- or EGFR-related effects of luteolin have been documented in a variety of tumors such as breast cancer, pancreatic cancer, and esophageal cancer, its direct mechanism of action and in vivo antitumor efficacy in cSCC remain lacking in systematic investigation [27]. Given that cSCC is characterized by ultraviolet-driven pathogenesis, a high mutational burden, and unique molecular evolutionary features, its signaling dependence and drug sensitivity may differ from those of other solid tumors [28,29]. Therefore, clarifying whether luteolin can inhibit the activation of the EGFR/PI3K/AKT pathway in A431 cells and further validating its antitumor effects in vivo is worthy of in-depth exploration.

## 2. Materials and Methods

### 2.1. Chemicals and Animals

The chemicals and materials used in this experiment are as follows: 5-Fluorouracil (5-Fu) (99.9%, CAS: 51-21-8, C13964619, Macklin, Shanghai, China); Luteolin (≥98%, RP210105, Madsen, Chengdu, China); 7,12-Dimethylbenz[a]anthracene (DMBA) (98%, A807576, Alphabio, Tianjin, China); Croton oil (≥95%, C865142, Macklin, Shanghai, China); high-glucose DMEM (2383696, Gibco, Waltham, MA, USA); fetal bovine serum (220315JF, Alphabio, Tianjin, China); DMSO (>99.9%, 20220301, Tianjin Hengxing, Tianjin, China); 4% paraformaldehyde tissue fixative (23039118, Beijing Lanjieke, Beijing, China); CCK-8 kit (101824133EF5E, Apexbio, Houston, TX, USA); Annexin V-FITC/PI apoptosis kit (2306001, Solarbio, Beijing, China); RIPA lysis buffer (IN-WB001, Invent Biotechnologies, Eden Prairie, MN, USA); Protease and Phosphatase Inhibitor Cocktail (051823230704, Beyotime, Shanghai, China); BCA protein assay kit (BL521A, Beyotime, Shanghai, China); DAB chromogenic kit (FL-6001, Changdao Biotech, Shanghai, China); Trizol reagent (1B14KA7304; Sangon Biotech, Shanghai, China); HiFiScript cDNA Synthesis Kit (26323, CWBio, Taizhou, China); anti-PI3K (1:1000, bs-2067R, Bioss, Beijing, China); anti-p-PI3K (1:1000, bs-5570R, Bioss, Beijing, China); anti-AKT (1:1000, bsm-33278M, Bioss, Beijing, China); anti-p-AKT (1:1000, bs-2720R, Bioss, Beijing, China); anti-EGFR (1:1000, bsm-33050M, Bioss, Beijing, China); anti-p-EGFR (1:1000, bsm-52149R, Bioss, Beijing, China); anti-BAX (1:1000, #2772, CST, Danvers, MA, USA); anti-Bcl-2 (1:1000, RO62O1556, Shenyang Wanyu, Shenyang, China); anti-β-actin (1:2000, AF2811, Beyotime, Shanghai, China); HRP-conjugated goat anti-rabbit IgG (1:8000, 234750818, ZSGB-BIO, Beijing, China); HRP-conjugated goat anti-mouse IgG (1:8000, 235680828, ZSGB-BIO, Beijing, China). The animals used were SPF grade BALB/c mice, weighing 20 ± 2 g, male, purchased from Liaoning Changsheng, after quality testing by Liaoning Provincial Laboratory Animal Quality Inspection Center; the result was qualified, and the license number was SCXK(Liao)-2020-0001. This experiment passed the qualification approval of the Experimental Animal Ethics Committee of Harbin University of Commerce (HUC EAEC, Approval No.: HSDYXY-2022014), and was performed in strict accordance with the ARRIVE 2.0 guidelines.

### 2.2. Cell Culture

Human skin squamous cell carcinoma A431 cells were obtained from the Beijing Union Medical College Cell Resource Center. The cells were cultured as a monolayer in DMEM supplemented with 10% FBS and antibiotics at 37 °C and in a 5% CO_2_ atmosphere.

### 2.3. Preparation of Luteolin Stock Solution

A quantity of 5.72 mg of luteolin powder was accurately weighed and fully dissolved in 200 μL of DMSO by vortexing and ultrasonication to prepare a 100 mmol/L stock solution. The prepared stock solution was stored at 4 °C in the dark until use. Subsequent working concentrations were freshly diluted with culture medium, and the final concentration of DMSO in all experimental groups was maintained at a non-toxic level to eliminate solvent interference.

### 2.4. CCK-8 Experiment

Logarithmic-phase A431 cells were harvested and resuspended as a single-cell suspension (5 × 10^4^ cells/mL) in complete DMEM, seeded into 96-well plates, and cultured for 24 h. The cells were then treatment with luteolin at concentrations of 20, 40, 60, 80, and 100 μmol/L for 24 h. After drug treatment, 10% CCK-8 working reagent was dispensed into each well, followed by a 0.5 h incubation, and absorbance (OD) values were measured at 450 nm. Each assay was performed with three biological replicates.

### 2.5. Annexin V-FITC/PI Apoptosis Assay

Logarithmic growth phase A431 cells were seeded into Petri dishes at an appropriate density. After 24 h, when cell density reached approximately 70–80%, the cells were treated with luteolin-containing medium at concentrations of 12, 24, and 48 μmol/L for 24 h. Following treatment, the cells were gently washed 2–3 times with PBS solution. EDTA-free 0.25% trypsin was added per well for digestion, followed by centrifugation. The pellet was resuspended in ice-cold PBS for counting, then centrifuged again, and the cell concentration was adjusted with 1× binding buffer. Aliquots of suspension were mixed with Annexin V-FITC and PI and incubated under light-protected conditions. Subsequently, PBS was added, and the samples were immediately analyzed. Cell apoptosis was detected by flow cytometer (Guava easyCyte 5; Millipore, Billerica, MA, USA) using Annexin V-FITC/PI double staining. All flow cytometric data were analyzed using FlowJo software (Version 10.8.1, Becton Dickinson & Company (BD), Franklin Lakes, NJ, USA). The fluorescence parameters of FITC (channel FL1) and PI (channel FL3) were collected, and forward scatter (FSC) and side scatter (SSC) were used to characterize cell size and granularity, respectively. The total apoptosis rate was calculated as the sum of the proportions of early and late apoptotic cells. All experiments were performed independently in triplicate.

### 2.6. qRT-PCR Assay

A431 cells in the logarithmic growth phase were seeded into 6-well plates at 2 × 10^5^ cells/well and cultured at 37 °C with 5% CO_2_ for 24 h. At 70–80% confluence, cells were treated with 12, 24, and 48 μmol/L luteolin for 24 h. Total RNA was extracted from the cells using TRIzol reagent. The cell apoptosis signaling pathway-related genes *EGFR*, *PI3K*, *AKT* and downstream genes *BAX* and *Bcl-2* were selected for study. The selected internal reference gene was the *β-actin* gene. Primers were designed using the online primer design tool of Sangon Biotech, and the amplified sequences are listed in Table 1. Total RNA concentration and purity were measured using a NanoDrop 2000 spectrophotometer (Thermo Fisher Scientific, Waltham, MA, USA). The sample with the lowest RNA concentration was used as the unified standard for subsequent reactions. cDNA was synthesized from 1 μg of total RNA using the HiFiScript cDNA Synthesis Kit (26323, CWBio, Taizhou, China) according to the manufacturer’s instructions. qRT-PCR reactions were performed using SYBR Green qPCR Mix (102122230407, Beyotime, Wuhan, China) on a QuantStudio™ 1 Plus Real-Time PCR System (Applied Biosystems, Waltham, MA, USA). The 20 μL reaction mixture contained 10 μL of SYBR Green Master Mix, 1 μL of forward primer (10 μM), 1 μL of reverse primer (10 μM), 2 μL of cDNA template, and 6 μL of nuclease-free water. The PCR cycling conditions were as follows: initial denaturation at 95 °C for 2 min, followed by 40 cycles of 95 °C for 15 s and 60 °C for 30 s. Melting curve analysis was performed to confirm amplification specificity. All reactions were performed in triplicate. Importantly, the change in the relative mRNA expression level of the target gene was calculated using the formula: 2 ^−ΔΔCT^.

### 2.7. Western Blot Assay

When the cell density reached about 70%, 12, 24, and 48 μmol/L luteolin-containing medium were added for 24 h. Cells were lysed on ice using RIPA lysis buffer supplemented with protease and phosphatase inhibitors. After centrifugation, the supernatant was collected to prepare protein samples. The protein concentration was determined using a BCA protein quantification kit, and all samples were adjusted to the same protein concentration. Proteins were separated by gel electrophoresis and then electrotransferred onto PVDF membranes under ice-bath conditions. After blocking with 5% skimmed milk, the membranes were incubated with primary antibodies overnight at 4 °C. Following three washes with TBST buffer, the membranes were incubated with secondary antibodies at room temperature for 1 h, and then washed three times again with TBST buffer. The protein blots were visualized using the Typhoon FLA9500 Multifunctional Biomolecular Imager (Cytiva, Marlborough, MA, USA). The gray values were finally quantified using ImageJ 1.46r software. The original uncropped blots are presented in the Supplementary Materials.

### 2.8. Molecular Docking

The three-dimensional crystal structure of the target protein was retrieved from the RCSB Protein Data Bank (RCSB PDB, https://www.rcsb.org/) (accessed on 10 April 2026), Its sequence integrity, functional domains, and active site information were validated using the UniProt database (https://www.uniprot.org/) (accessed on 10 April 2026). Protein pretreatment was performed using AutoDock Tools 1.5.7 software. The pretreatment procedure included removal of crystallized water molecules, co-crystallized native ligands, and non-specifically bound small molecules from the protein structure, followed by the addition of polar hydrogen atoms and calculation of Gasteiger charges. The pretreated protein was subsequently converted into PDBQT format for subsequent docking analysis. The two-dimensional structure of luteolin was obtained from the PubChem database (https://pubchem.ncbi.nlm.nih.gov/ ) (accessed on 10 April 2026). After three-dimensional conformation optimization, charge calculation, and rotatable bond assignment, luteolin was converted into PDBQT format to serve as the ligand for docking. Molecular docking simulation was conducted using AutoDock Tools with the docking region targeting the functional active pocket of the target protein. The binding affinity between the ligand and the receptor was evaluated by the binding free energy. PyMOL 2.6.0 software was used for three-dimensional visualization of the optimal binding conformation, as well as the analysis of the binding mode and key intermolecular interactions between luteolin and the target protein.

### 2.9. Animal Experiment

SPF-grade experimental mice were acclimated for 7 days under constant temperature, constant humidity, and a 12-h light/dark cycle. Three days before the experiment, an area of approximately 2 × 2 cm on the back of each mouse was shaved with a disposable razor. During the experiment, hair was shaved at irregular intervals depending on hair regrowth. In the first three weeks of the experiment, 200 μL of 0.75 mg/mL DMBA acetone solution was applied topically to the shaved area once a week (the blank control group received an equal volume of acetone solution), and from the fifth week onwards, 200 μL of 0.25% croton oil acetone solution was applied to the same area twice weekly (the blank control group was applied with an equal volume of acetone solution), and this treatment continued for 12 weeks. The total experimental duration was 16 weeks. The mice were randomly assigned to the blank control group, DMBA-induced model group, luteolin group, and 5-FU positive control group based on their initial body weight using a random number table, with 10 mice in each group (*n* = 10), for a total of 40 mice. The sample size was established by referring to the standard methodologies employed in comparable in vivo studies [30], while strictly adhering to the 3R principles (Replacement, Reduction, Refinement) for animal research. From week 5 until the end of the experiment, mice in the Control group and DMBA group received daily oral gavage of PBS solution at a standardized volume of 0.1 mL per 10 g body weight. Mice in the Luteolin group were given daily intragastric luteolin at 60 mg/kg body weight (corresponding to a fixed volume of 0.1 mL/10 g body weight), and the administration lasted continuously until the 16th week of the experiment. All outcome assessments were performed in a single-blinded manner by independent researchers who were unaware of the group allocation. Unblinding was conducted only after all data collection and statistical analysis were completed.

#### 2.9.1. Mouse Weight Measurement

Throughout the experiment, mice were weighed weekly using an electronic balance to monitor weight fluctuations. Body weights for each group were expressed as the mean ± standard deviation of 10 mice in the group.

#### 2.9.2. Determination of Tumor Nodules Inhibition Rate

By week 16, tumor nodule counts and survival rates of mice across all experimental groups were summarized, and the mean tumor nodule number for each mouse group was then calculated. By comparing the mean tumor nodule count of each treatment group with that of the control group, the relative percentage deviation from the control group was computed to ascertain the tumor nodule inhibition rate.

(1) Tumor nodule inhibition rate (%) = [(Mean number of nodules in control group − Mean number of nodules in treatment group)/Mean number of nodules in control group] × 100%

#### 2.9.3. Immune Organ Index

After the termination of all experimental procedures, blood was collected from the retro-orbital sinus of the mice, after which the mice were euthanized by cervical dislocation. The thymus and spleen were then removed, weighed using an analytical balance, and the immune organ index was calculated as follows:

(2) Organ coefficient = organ weight (mg)/animal net weight (g)

#### 2.9.4. Skin Tissue Sampling

After 16 weeks, mouse skin samples were collected for tissue analysis. The detailed sampling procedure was as follows: The mice were euthanized, and the tumor-bearing skin on the back was removed with sterilized ophthalmic scissors and tweezers, and the subcutaneous fat was removed. Next, the isolated skin tissue was trimmed into rectangles of appropriate dimensions, and placed on the prepared aluminum foil with the dermis side facing downward. The skin tissue was when gently smoothed and fully spread out with tweezers, and the surrounding tin foil was folded along the contour of the skin tissue to immobilize the sample; this step served to prevent the skin from curling during subsequent processing, which might otherwise compromise accuracy of tissue analysis results. The wrapped tissue samples were then immersed in neutral formalin fixative for preservation. After fixing at room temperature for 24 h, the skin tissue was removed and transferred to a plastic embedding box, and then re-immersed in neutral formalin fixative for subsequent experiments.

#### 2.9.5. HE Staining

The skin pathological morphology of mice in each group was compared using hematoxylin and eosin staining (HE staining) and microscopic examination. The experimental steps were as follows: The sections were baked at 60 °C for 30 min, then treated with xylene I and xylene II, each for 10 min for dewaxing; subsequently, the sections were sequentially incubated with 100%, 95%, 85%, and 75% ethanol solutions and double distilled water, each for 3 min. They were then stained with hematoxylin aqueous solution, followed by bluing in ammonia water; after rinsing under running water for 1 h, the sections were immersed in 70% and 90% ethanol for 10 min each, then stained with alcoholic eosin for 2 min, cleared with xylene, and finally mounted with neutral gum mounting medium.

#### 2.9.6. Ki-67 Immunohistochemistry

The experimental steps for immunohistochemistry were as follows: The sections were baked at 60 °C for 30 min, then treated with xylene I and xylene II for 10 min each to deparaffinize. Subsequently, the sections were processed with a graded series of 100%, 95%, 85%, and 75% ethanol solutions followed by double-distilled water, with each treatment lasting 3 min. Subsequently, the tissue sections were subjected to antigen retrieval under high-pressure conditions for 20–30 min. After washing three times with 0.02 M PBS (3 min each), the sections were incubated in a wet box with 3% H_2_O_2_ for 10 min. Following another PBS wash, non-immune normal sheep serum was added dropwise, and the sections were incubated in a wet box at 37 °C for 30 min, then washed in PBS, and the primary Ki-67 rabbit monoclonal antibody (Clone number: D3B5, Catalog No. #9027, Cell Signaling Technology, Danvers, MA, USA) was incubated at a dilution ratio of 1:200 overnight at 4 °C. The sections were removed and placed at room temperature for 40 min and washed in PBS, and the HRP-conjugated broad-spectrum secondary antibody (D-3004, Shanghai Changdao Biotechnology, Shanghai, China), at a dilution ratio 1:500, was incubated for 60 min at 37 °C. PBS washing, DAB staining, running water rinsing, hematoxylin counterstaining, and 0.1% hydrochloric acid alcohol differentiation were carried out, followed by ethanol solution gradient dehydration, xylene transparent treatment, and neutral resin mounting. Ki-67-positive expression area was quantified using ImageJ software (National Institutes of Health, Bethesda, MD, USA), with 15 randomly selected non-overlapping high-power fields per group for %Area value calculation.

### 2.10. Data Statistics

Most figures included in this study were generated via GraphPad Prism 9.5. SPSS 26.0 was utilized to determine the homogeneity of variance of the experimental outcomes. The experimental data were as follows: $\bar{x}$ ± *s*. One-way ANOVA was performed for intergroup differences, with Tukey’s post hoc test for pairwise comparisons; *p* < 0.05 was statistically significant.

## 3. Results

### 3.1. Effect of Luteolin on the Activity of A431 Cells

**Figure 2:**
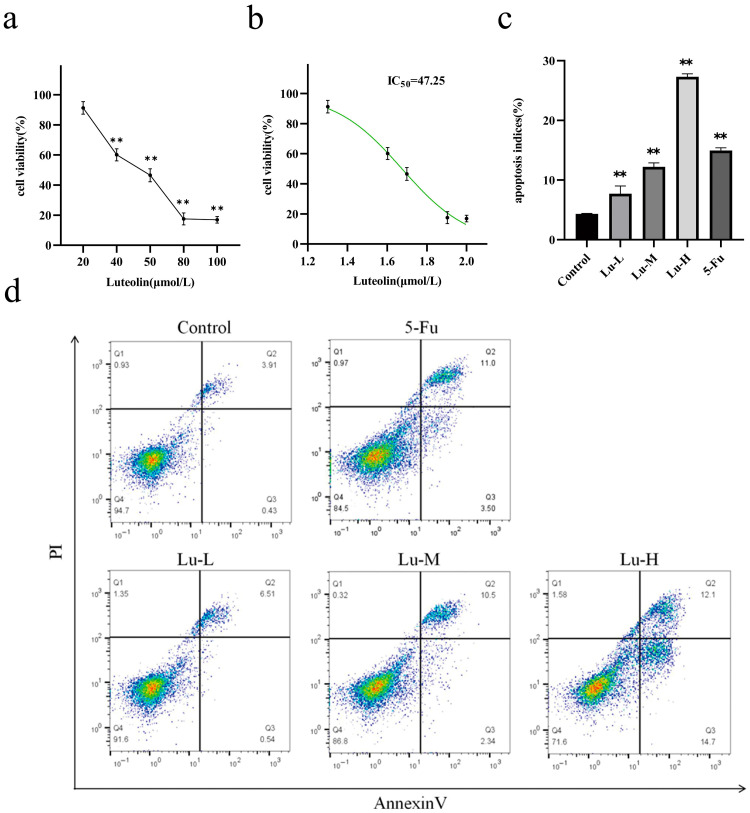
Effect of luteolin on the viability and apoptosis of A431 cells. (**a**) Cell viability curve. (**b**) Fitted curve. (**c**) Effect of different luteolin concentrations on apoptosis of A431 cells after treatment for 24 h. (**d**) Statistical histogram of apoptosis index (*n* = 3). Lu-L, Lu-M, and Lu-H represent the low-, medium-, and high-dose luteolin groups, respectively. ** *p* < 0.01 compared to the control group.

The effect of luteolin on A431 cell viability after 24 h of treatment was evaluated using the CCK-8 assay. The cell viability curve and fitted curve are presented in Figure 2a,b, respectively, and the IC_50_ value of A431 cells was calculated. The results showed that luteolin exerted a significant inhibitory effect on A431 cell viability in a dose-dependent manner (*p* < 0.05). The IC_50_ of luteolin in A431 cells was 48 μmol/L; accordingly, concentrations of 12, 24, and 48 μmol/L were selected as the working doses for subsequent experiments.

### 3.2. Effect of Luteolin on Apoptosis of A431 Cells

Apoptosis was assessed by flow cytometry following Annexin V-FITC/PI double staining. In the resulting dot plots, cells were classified into four populations based on their fluorescence intensity: necrotic or dead cells (Annexin V^−^/PI^+^, Q1), late apoptotic cells (Annexin V^+^/PI^+^, Q2), early apoptotic cells (Annexin V^+^/PI^−^, Q3), and viable cells (Annexin V^−^/PI^−^, Q4). The corresponding results are shown in Figure 2c,d. In the control group, the total apoptosis rate was 4.34 ± 0.07%, consisting of 3.86 ± 0.06% late apoptotic cells and 0.56 ± 0.04% early apoptotic cells, which represented the basal apoptosis level of the cells. Compared with the blank control group, luteolin at concentrations of 12 μmol/L, 24 μmol/L, and 48 μmol/L significantly increased the apoptosis rate of A431 cells (*p* < 0.01) in a concentration-dependent manner, with values of 7.7 ± 1.31%, 12.22 ± 0.66%, and 27.3 ± 0.5%, respectively. The apoptosis rate in the 5-Fu group was 14.96 ± 0.47%, including 3.72 ± 0.22% early apoptotic cells and 11.23 ± 5.81% late apoptotic cells. Notably, the apoptosis level induced by 48 μmol/L luteolin was superior to that induced by 80 μmol/L 5-Fu, which was mainly attributed to the marked increase in early apoptotic cells under luteolin treatment, reaching 14.67 ± 0.15%. Following luteolin intervention, the proportion of necrotic cells was less than 2%. This finding suggests that luteolin induces controlled programmed cell death in A431 cells rather than necrosis, exhibiting a specific and potent pro-apoptotic effect.

### 3.3. Effect of Luteolin on the Expression of A431 Cell-Related Genes

**Figure 3:**
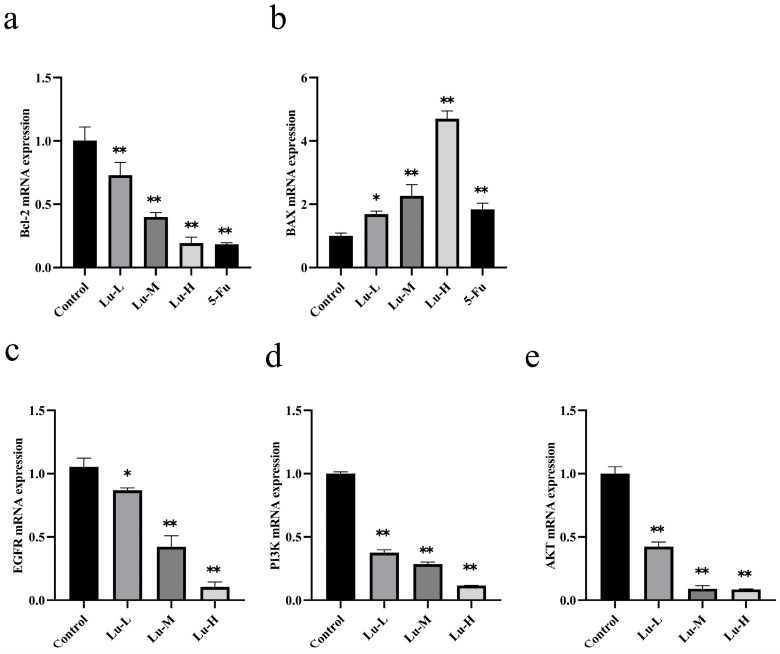
Effect of Luteolin/5-Fu on apoptosis-related genes in A431 cells. (**a**) *Bcl-2*; (**b**) *BAX*; (**c**) *EGFR*; (**d**) *PI3K*; (**e**) *AKT* mRNA levels. *n* = 3. * *p* < 0.05, ** *p* < 0.01 vs. control group.

Relative to the control group, luteolin exerted a significant dose-dependent effect on A431 cells: *Bcl-2* mRNA expression was markedly downregulated while *BAX* significantly upregulated (*p* < 0.01, Figure 3a,b).

As depicted in Figure 3c–e, versus the control group, luteolin treatment resulted in a significant reduction in *EGFR*, *PI3K*, and *AKT* mRNA levels (*p* < 0.05); Notably, this downregulatory effect was more pronounced in the medium and high-dose luteolin groups (*p* < 0.01). These findings demonstrate that luteolin can inhibit the mRNA expression of key genes in the EGFR/PI3K/AKT signaling pathway.

### 3.4. Effect of Luteolin on the Expression of A431 Cell-Associated Protein

**Figure 4:**
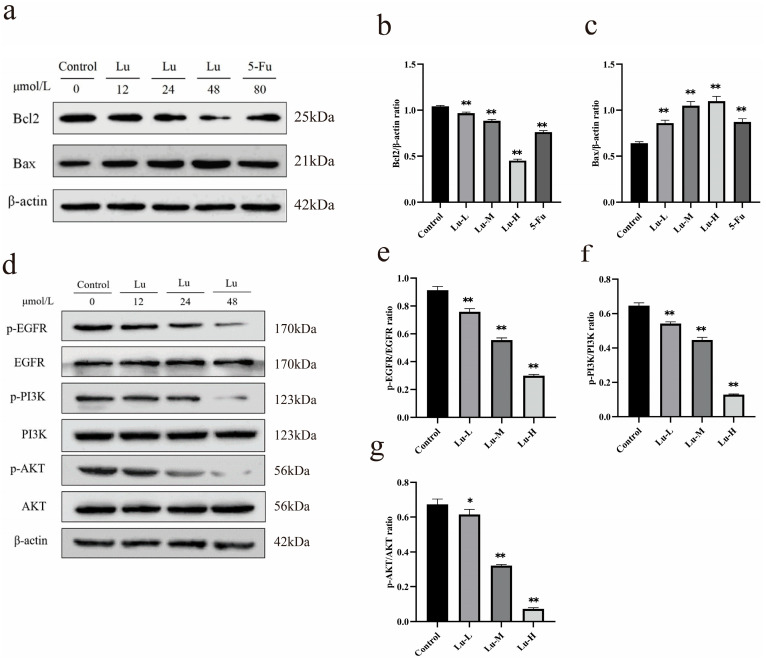
Effect of luteolin/5-Fu on apoptosis-related proteins of A431 cell. (**a**) Bcl-2 and Bax protein expression (luteolin/5-Fu treatment); (**b**) Bcl-2/β-actin ratio; (**c**) Bax/β-actin ratio; (**d**) EGFR, PI3K, AKT, and phosphorylated protein levels; (**e**) p-EGFR/EGFR ratio; (**f**) p-PI3K/PI3K ratio; (**g**) p-AKT/AKT ratio. * *p* < 0.05, ** *p* < 0.01 vs. control group.

Upon exposure to 12, 24, and 48 μmol/L luteolin, the protein level of Bax was markedly upregulated, while that of Bcl-2 was dose-dependently downregulated in A431 cells (*p* < 0.05, Figure 4a–c).

The effects of luteolin on the key proteins in this signaling pathway, p-EGFR, EGFR, p-PI3K, PI3K, p-AKT, and AKT, were tested by applying Western blot technology. As shown in Figure 4d–g, luteolin decreased p-EGFR, p-PI3K, and p-AKT, while the expression of EGFR, PI3K, and AKT did not significantly change with increased concentration, so p-EGFR/EGFR, p-PI3K/PI3K, p-AKT/AKT were significantly reduced (*p* < 0.05). The results indicate that luteolin inhibits pathway protein phosphorylation and exerts anti-cancer effects on A431 cells by inhibiting the EGFR/PI3K/AKT pathway.

### 3.5. Molecular Docking Analysis

**Figure 5:**
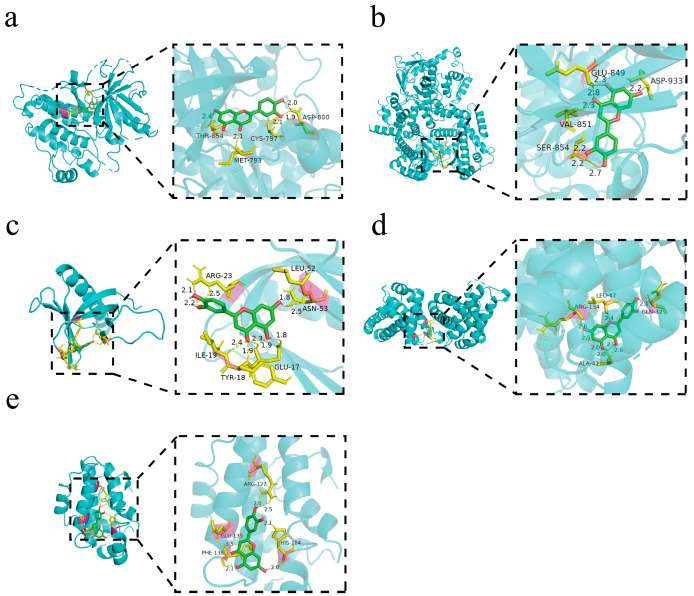
Molecular docking analysis of luteolin with EGFR, PIK3CA, AKT, Bax, and Bcl-2. (**a**) EGFR; (**b**) PIK3CA; (**c**) AKT; (**d**) Bax; (**e**) Bcl-2.

The PDB identification codes of the target proteins involved in this study are as follows: EGFR (PDB: 2RGP), Akt (PDB: 1UNP), PIK3CA (PDB: 8BFU), Bcl-2 (PDB: 6O0K), and Bax (PDB: 4S0O). Molecular docking analysis revealed that luteolin exhibited binding activity toward EGFR, PIK3CA, AKT, Bax, and Bcl-2 (Figure 5a–e). The minimum binding energies of luteolin with EGFR, PIK3CA, AKT, Bax, and Bcl-2 were −5.41, −5.52, −5.83, −6.87, and −5.87 kcal/mol, respectively. Specifically, luteolin formed hydrogen bonds with ASP-800, CYS-797, MET-793, and THR-854 in EGFR; GLU-849, ASP-933, VAL-851, and SER-854 in PIK3CA; APG-23, LEU-52, ASN-53, ILE-19, TYR-18, and GLU-17 in AKT; LEU-47, ARG-134, GLN-32, and ALA-42 in Bax; and ARG-127, HIS-184, GLU-135, PHE-138 in Bcl-2. Among all detected targets, luteolin showed the lowest binding energy with Bax, suggesting a relatively stronger binding tendency. Collectively, these results provide structural evidence supporting that luteolin may interact with EGFR/PI3K/AKT signaling-related proteins and apoptosis-associated proteins.

### 3.6. Establishment of cSCC Mouse Model and Luteolin Efficacy

#### 3.6.1. Mouse Body Weight Measurement

**Figure 6:**
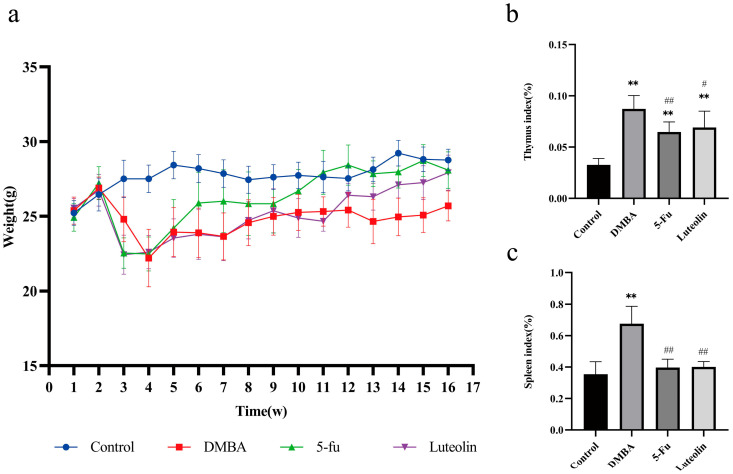
Diagram of mouse coat, body weight signs, and immune index. (**a**) Observation of mice. (**b**) Thymus indexes. (**c**) Spleen indexes (*n* = 10). ** *p* < 0.01 vs. control group; # *p* < 0.05, ## *p* < 0.01 vs. DMBA group.

As shown in Figure 6a, the mean body weight of mice in the control group showed a steady increasing trend throughout the experimental period. During the first four weeks, body weight in the remaining groups (including the DMBA-induced cSCC model group and all luteolin-treated groups) declined to varying extents. This result indicates that the DMBA-induced cSCC model developed tumor-associated cachexia accompanied by obvious body weight loss. Starting from the sixth week, following continuous luteolin administration, the body weight of mice in each treated group gradually recovered in recovery amplitude. In comparison, mice in the DMBA model group presented severe emaciation, and their body weight growth gain was markedly lower than that of the control group, indicating progressive deterioration of the cachectic status.

#### 3.6.2. Assessment of the Tumor Nodule Inhibitory Rate

The inhibition rate of tumor nodules determines the impact of impeding tumor development of cSCC in mice in each administration group, as shown in Table 2. After luteolin administration, the occurrence and development of skin cancer in mice were inhibited, and the tumor nodule inhibition rate reached 45.28%, which had a good therapeutic effect.

#### 3.6.3. Changes in Immune Organ Index

The immune organ index was calculated as the ratio of thymus and spleen weight to body weight, which can reflect the immune function and general health status of experimental animals. As shown in Figure 6b,c, the thymus index of mice in the blank control group was 0.033 ± 0.006%. In addition, the thymus index of model group mice was notably elevated, and the difference was statistically significant (*p* < 0.01). Compared with the model group, luteolin group and positive drug group intervention significantly reduced the thymus index and spleen index (*p* < 0.05).

#### 3.6.4. Histopathological Observation via HE Staining

**Figure 7:**
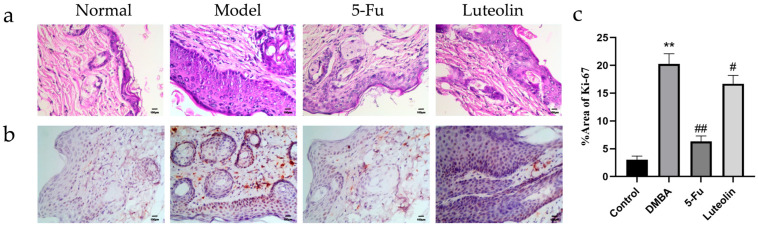
Tissue microscopic condition. (**a**) HE stains of mice skin tissues (200×). (**b**) Expression status of Ki-67 (200×). (**c**) Statistical chart of Ki-67 expression (*n* = 10). ** *p* < 0.01 vs. control group; # *p* < 0.05, ## *p* < 0.01 vs. DMBA group.

The positive expression area of Ki-67 in each group was quantitatively analyzed using ImageJ software. A total of 15 visual fields were randomly selected for each group to calculate the %Area value. According to the HE staining results in Figure 7a, the epidermis was normal, and the collagen fibers were almost parallel to the skin. The hair follicles and sweat glands were clearly visible. In contrast, in the model group, the skin was markedly thickened, and cutaneous appendages such as sweat glands were remarkably reduced. Focal squamous hyperplasia of small proliferative cells was observed in tumorous lesions; hair follicles remained relatively intact with regular cellular arrangement.

#### 3.6.5. Assessment of Tumor Cell Proliferative Activity via Ki-67 Immunohistochemical Staining

Immunohistochemistry was performed on skin tissue to clarify the inhibitory effect of monomers on tumor proliferation in the mouse cSCC models. Ki-67 can be used to indicate cell proliferative activity. Cells with clear brown-yellow staining in the nucleus were defined as Ki-67-positive cells. Data from Figure 7b,c revealed that compared with the control group, the expression of Ki-67—a key proliferation marker—in epithelial cells of the DMBA-treated group was markedly upregulated. Numerous brown particles were visible, indicating that the cells region underwent abnormal proliferation and differentiation. Except for the positive drug group, the Ki-67 value of the monotherapy group was markedly lower than those detected in the DMBA group (*p* < 0.05).

## 4. Discussion

Globally, skin cancer is one of the most prevalent malignancies, affecting individuals of different ethnicity and from various regions. Among skin cancers, cSCC is the second most prevalent form of skin cancer, exhibiting high invasiveness and a significant rate of recurrence. It predominantly occurs on the head and face; the pathological manifestations of cSCC are persistent, independent hard papules or red nodules that may bleed spontaneously, as well as superficial ulcers with pus discharge [31,32]. In the early stages, surgery is the preferred treatment, although it often results in large wounds. For mid-to-late-stage and invasive squamous cell carcinoma, a combination of chemotherapy and other therapies is used alongside surgery [33], but the effect is still not ideal, the side effects are large, and drug resistance is easy to develop, which brings great mental and economic pressure to patients. Therefore, discovering effective and safe natural therapies for cSCC is of practical significance for tumor prevention and treatment, prolonging patient survival, assisting traditional treatment methods, and supplementing combined medication.

Huang et al. [34] demonstrated that luteolin impairs the proliferative activity of MDA-MB-231 breast cancer cells by inducing arrest in the S-phase cell cycle arrest. This effect occurs through the downregulation of hTERT expression, which leads to decreased telomerase activity. Separately, luteolin has also been shown to inhibit the expression of MMP-2 and MMP-9 in A431 cells, thereby exerting potent antitumor activity [27].

Considering the antitumor properties of luteolin, we initially evaluated its impact on the viability of human A431 cells in vitro using the CCK-8 assay. The findings demonstrated that luteolin reduced A431 cell viability in a concentration-dependent manner. Flow cytometry analysis further confirmed that luteolin increased the percentage of apoptotic A431 cells in a concentration-dependent manner. Programmed cell death encompasses multiple modalities, with apoptosis representing one of them. Its abnormal regulation can lead to a variety of diseases. For instance, excessive apoptosis is closely linked to neurodegenerative and autoimmune diseases, whereas inadequate apoptosis enables tumor cells to evade the immune system and develop drug resistance, leading to cancer [35,36]. Therefore, inducing apoptosis is an effective method for treating cancer. It exerts its effects in a flow cytometry-detectable manner. Its cytotoxic effect on A431 cells may partially depend on the induction of apoptosis.

Although luteolin’s antitumor effects have been validated in multiple cancers by regulating apoptosis and cell cycle arrest, its specific regulatory mechanisms in cSCC cells (e.g., A431 cells) and in vivo cSCC models remain to be fully elucidated, especially with respect to the EGFR/PI3K/AKT signaling pathway and Bcl-2/Bax-mediated apoptotic cascade. The EGFR/PI3K/AKT signaling pathway is involved in maintaining cellular homeostasis and regulating cell growth, differentiation, migration, and apoptosis. It serves as a critical molecular mechanism underlying the occurrence and development of head and neck squamous cell carcinoma [37]. Accumulating studies have indicated that EGFR is overexpressed in cSCC, which triggers cell cycle disorders, excessive anti-apoptotic signals, and uncontrolled cell growth, thereby promoting the initiation and progression of cSCC [38]. To gain deeper insights into how luteolin induces A431 cell apoptosis, we performed experimental validation using qRT-PCR and Western blot techniques. Our findings demonstrated that luteolin modulates the expression of apoptotic regulatory genes by downregulating *Bcl-2* and upregulating *BAX* at both mRNA and protein levels. Furthermore, luteolin inhibited *EGFR*, *PI3K*, and *AKT* mRNA expression and protein phosphorylation, indicating that luteolin may may be related to the EGFR/PI3K/AKT signaling pathway and thereby trigger A431 cell apoptosis.

Molecular docking was further performed to explore the potential interactions between luteolin and EGFR/PI3K/AKT signaling-related proteins as well as apoptosis-associated proteins. The docking results suggested that luteolin could stably bind within the binding pockets of EGFR, PIK3CA, AKT, Bax, and Bcl-2, forming hydrogen bonds and polar interactions with multiple amino acid residues in the simulated molecular environment. It should be clarified that, as an auxiliary virtual prediction method, molecular docking cannot confirm the direct binding between luteolin and the above proteins in a biological context, and the results are only used as preliminary theoretical reference. Combined with the reduced phosphorylation of EGFR, PI3K and AKT, as well as the altered expression of Bax and Bcl-2 observed in our experiments, these in silico data provide a potential molecular-level explanation for the regulatory effect of luteolin on EGFR/PI3K/AKT signaling and apoptosis-related pathways, which may be associated with its anti-cSCC activity.

Based on the inhibitory effect of luteolin on cutaneous squamous cell carcinoma cells in vitro, we further investigated its in vivo antitumor efficacy using a two-stage skin carcinogenesis mouse model induced by DMBA and croton oil. The chemical induction method is currently the most commonly used experimental mouse cSCC model [39]. Compared with the subcutaneous xenograft tumor models, the chemical induction method closely mimics the pathogenesis of human cSCC after exposure to chemical carcinogens, and intuitively shows the process of skin ulceration, hyperplasia, and malignant tumor growth. At the endpoint of the experiment, all the model group mice survived with no mortality observed. The tumor incidence rate was 0% in the control group and 100% in the model group. The tumor characteristics of the model group, together with the cSCC-typical histopathological features revealed by H&E staining, confirmed that the model was successfully established. The doses of luteolin were selected based on previously reported in vivo antitumor efficacy and safety data, while also considering the exploratory nature of this study and animal welfare requirements. A quantity of 150 mg/kg oral luteolin suppressed A549 non-small cell lung cancer xenograft growth, while 50–200 mg/kg dosing inhibited tumor progression and enhanced antitumor immunity in H22 hepatoma-bearing mice. The 60 mg/kg dose in this study lies within this validated active range, and was thus chosen as a moderate oral intervention to assess luteolin’s in vivo anti-cutaneous squamous cell carcinoma effect [40,41]. A single fixed dose simplified experimental variables, avoided unnecessary interference, and conformed to the 3R principle to reduce animal consumption.

In vivo experiments have proven that luteolin can improve the skin disease status and weight loss caused by cSCC in mice, and that luteolin can enhance the tumor nodule inhibition rate and reduce the thymus and spleen index, suggesting that luteolin may slow down tumor occurrence and development. The nuclear protein Ki-67 is commonly used as a marker of cellular malignancy and is actively expressed in G1, S, G2, and M phases but not in the G0 (resting) phase, and is upregulated in hyperproliferative inflammatory skin lesions as well as in cutaneous malignancies [42,43]. The expression level of Ki-67 is closely associated with tumor proliferative activity and histopathological aggressiveness in cSCC [44]. Compared with the control group, Ki-67-positive expression was significantly elevated in the model group, indicating abnormal proliferation in mouse epithelial cells. Luteolin treatment led to a pronounced reduction in Ki-67 expression compared with the model group (*p* < 0.01), highlighting that luteolin’s anti-cSCC activity in mice likely correlates with the inhibition of tumor cell proliferative activity.

Despite the favorable in vivo therapeutic effects of luteolin observed in the DMBA/croton oil-induced skin carcinogenesis model, several limitations should be acknowledged. First, only a single dose of luteolin was evaluated in the DMBA/croton oil-induced mouse model of cSCC, and a comprehensive dose–response assessment was not performed. This choice was primarily made to provide a proof-of-concept evaluation of the antitumor efficacy of luteolin in a classical chemical carcinogenesis model, with 5-Fu included as a positive control to validate the responsiveness of the experimental system. Consequently, the optimal therapeutic dose and the potential dose-dependent effects of luteolin remain to be elucidated. Second, while the DMBA/croton oil-induced model is a well-established and clinically relevant cSCC model, it does not fully recapitulate all aspects of human cSCC, particularly its genetic heterogeneity and the complexity of the tumor microenvironment. Third, the activation of the EGFR/PI3K/AKT pathway was preliminarily inferred from in vitro data; the expression and phosphorylation of pathway-related proteins were not validated in tumor tissues. Future studies are warranted to explore multiple dose regimens, employ additional cSCC models, and further verify the activation of this signaling pathway in vivo.

## 5. Conclusions

The novelty of this work lies in exploring luteolin’s dual role in suppressing cSCC cell proliferation and inducing apoptosis, as well as its regulation of intrinsic signaling pathways. Previous studies on luteolin in cSCC only focused on in vitro anti-proliferative activity, with in vivo efficacy and molecular mechanisms rarely explored. Our study addresses this gap via cellular and animal models, laying a foundation for luteolin as an adjuvant therapy for cSCC and a candidate for translational research. In conclusion, luteolin exhibits significant therapeutic potential for cSCC in both in vitro and in vivo models, and its underlying mechanism may be associated with the inhibition of tumor cell proliferation and the induction of apoptotic cell death. This study provides a solid experimental basis for further basic mechanistic research of luteolin against cSCC.

## Figures and Tables

**Table 1:** Primer sequence of apoptosis-related genes.

Primer Name	Sequences (5′ to 3′)	Number of Bases
Bcl-2(F)	TACGAGTGGGATGCGGGAGATG	22
Bcl-2(R)	CCGGGCTGGGAGGAGAAGATG	21
BAX(F)	GATGCGTCCACCAAGAAGCTGAG	23
BAX(R)	CACGGCGGCAATCATCCTCTG	21
EGFR(F)	GTGTGCCACCTGTGCCATCC	20
EGFR(R)	GCCACCACCAGCAGCAAGAG	20
PI3K(F)	ATGGTGAGGCGGAGGACAGTG	21
PI3K(R)	TGCTGTCGTTCGCTCATCATCAC	23
AKT(F)	GGTCATGCGCTTACGGAACA	20
AKT(R)	CGCGCACCGTAAAGTTGTTG	20
β-actin(F)	CCTGGACTTCGAGCAAGAGATGG	23
β-actin(R)	CAGGAAGGAAGGCTGGAAGAGTG	23

**Table 2:** Inhibition rate of tumor nodules.

Group	Surviving Mice	Tumor-Bearing Mice	Tumor Nodules	Average Number of Tumors per Mouse	Tumor Nodule Inhibition Rate (%)
Control	10	0	0	0	—
DMBA	10	10	53	5.3	—
5-Fu	10	9	20	2	62.26
Luteolin	10	9	29	2.9	45.28

## Data Availability

The original data presented in the study are included in the article, and the original data required for the patent application can be inquired to the corresponding author.

## Supplementary Materials

The following supporting information can be downloaded at: https://www.mdpi.com/article/10.3390/biom16050737/s1, Figure S1: Raw Western blot bands corresponding to Figure 4a; Figure S2: Raw Western blot bands corresponding to Figure 4d; Table S1: Quantitative analysis of relative expression levels of Bcl-2 and Bax in luteolin-treated A431 cells; Table S2: Quantitative analysis of relative expression levels of total and phosphorylated EGFR, PI3K and AKTinluteolin-treated A431 cells.
