# Supplementary material for: Potential Efficacy of Luteolin in Cutaneous Squamous Cell Carcinoma: A Combined In Vitro and In Vivo Study
Source: Biomolecules. 2026 May 18;16(5):737. doi: 10.3390/biom16050737 (PMC13204118; doi:10.3390/biom16050737)
Supplement: Supplementary file 1 [file biomolecules-16-00737-s001.zip › biomolecules-4294796-supplementary.pdf]

Potential Efficacy of Luteolin in Cutaneous Squamous Cell Carcinoma: A Combined *In Vitro* and *In Vivo* Study

Abstract: Cutaneous squamous cell carcinoma (cSCC) is a common malignant skin tumor with invasive potential and risk of recurrence. This study investigated the anti-cSCC effects of luteolin in vitro and in vivo and explored the associated molecular mechanisms. The effects of luteolin on A431 cell viability were assessed by CCK-8 assay, and apoptosis was analyzed by Annexin V-FITC/propidium iodide (PI) double staining. qRT-PCR and Western blot analyses were performed to evaluate apoptosis-related factors and the EGFR/PI3K/AKT signaling pathway. Molecular docking was further conducted to explore the potential interactions of luteolin with EGFR/PI3K/AKT signaling-related proteins and apoptosis-associated proteins. In vivo, a two-stage skin carcinogenesis model induced by 7,12-dimethylbenz[a]anthracene (DMBA) and croton oil was used to evaluate the antitumor activity of luteolin. Luteolin significantly inhibited A431 cell viability and promoted apoptosis in a concentration-dependent manner. Moreover, luteolin increased Bax expression and decreased Bcl-2 expression at both the mRNA and protein levels. Mechanistically, luteolin suppressed the phosphorylation of EGFR, PI3K, and AKT. Molecular docking suggested that luteolin could interact with EGFR, PIK3CA, AKT, Bax, and Bcl-2, providing supportive in silico evidence for its potential modulation of EGFR/PI3K/AKT signaling and apoptosis-related proteins. In vivo, luteolin alleviated body weight loss, achieved a tumor nodules inhibition rate of 45.28%, significantly improved spleen and thymus indices($p < 0.05$), and ameliorated histopathological damage in skin tissues. In addition, immunohistochemical analysis showed that luteolin reduced Ki-67 expression. These results indicate that luteolin exerts anti-cSCC effects in vitro and in vivo, possibly through modulation of the EGFR/PI3K/AKT signaling pathway and apoptosis-related proteins.

Keywords: Luteolin; cSCC; Apoptosis; A431; EGFR/PI3K/AKT

Supporting Information List

Raw Western blot bands and quantitative analysis data for Figure 4

Figure 4a：Effects of luteolin on apoptotic proteins in A431 cells

Bax (left→right): control, Luteolin (12, 24, 48μM), 5-Fu

Bcl2 (left→right): control, Luteolin (12, 24, 48μM), 5-Fu

β-actin (left→right): control, Luteolin (12, 24, 48μM), 5-Fu

Figure 4d：Effects of luteolin on EGFR/PI3K/AKT signaling pathway proteins in A431 cells

p-EGFR (left→right): control, Luteolin (12, 24, 48μM)

EGFR (left→right): control, Luteolin (12, 24, 48μM)

p-PI3K (left→right): control, Luteolin (12, 24, 48μM)

PI3K (left→right): control, Luteolin (12, 24, 48μM)

p-AKT (left→right): control, Luteolin (12, 24, 48μM)

AKT (left→right): control, Luteolin (12, 24, 48μM)

β-actin (left→right): control, Luteolin (12, 24, 48μM)

Figure S1: Raw Western blot bands corresponding to Figure 4a

Bax

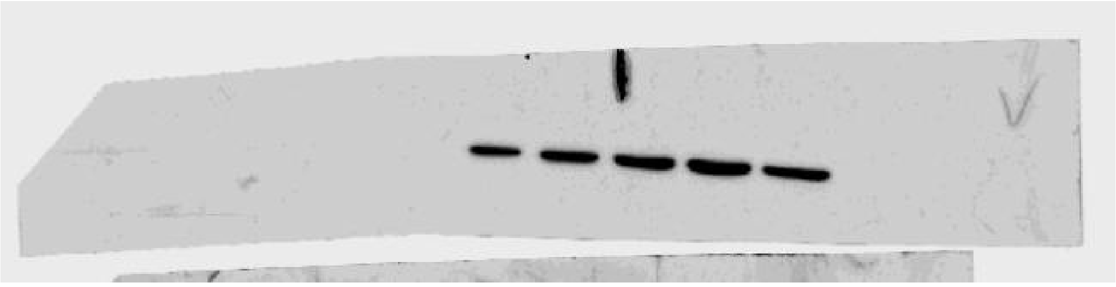

Bcl2

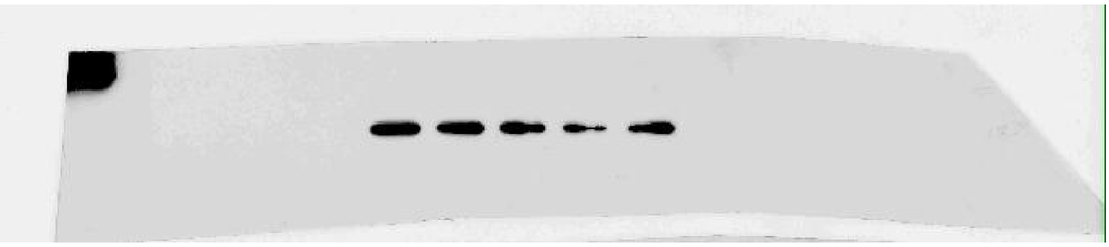

β-actin

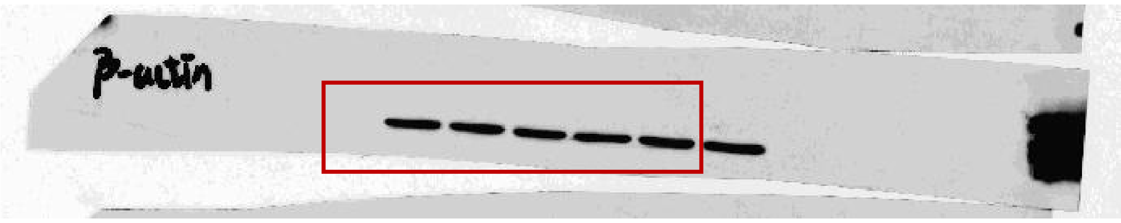

Table S1 Quantitative analysis of relative expression levels of Bcl-2 and Bax in luteolin-treated A431 cells

Group	Bcl2/β-actin	Bax/β-actin
Control	1.041±0.009	0.642±0.015
Lu-L	0.968±0.011	0.860±0.030
Lu-M	0.883±0.014	1.048±0.046
Lu-H	0.452±0.016	1.099±0.051
5-Fu	0.762±0.018	0.872±0.035

Figure S2: Raw Western blot bands corresponding to Figure 4d

P-EGFR

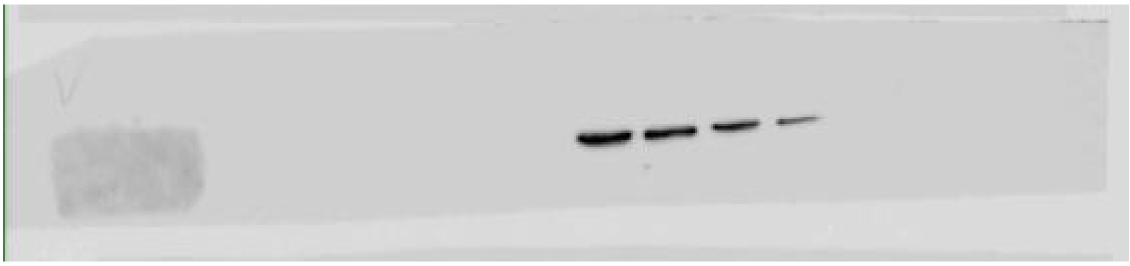

EGFR

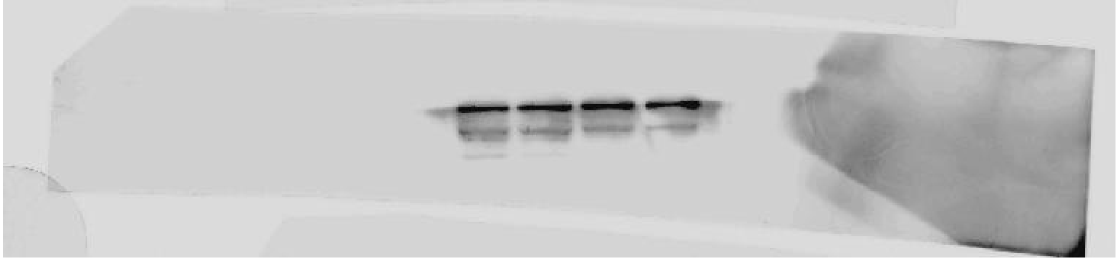

P-PI3K

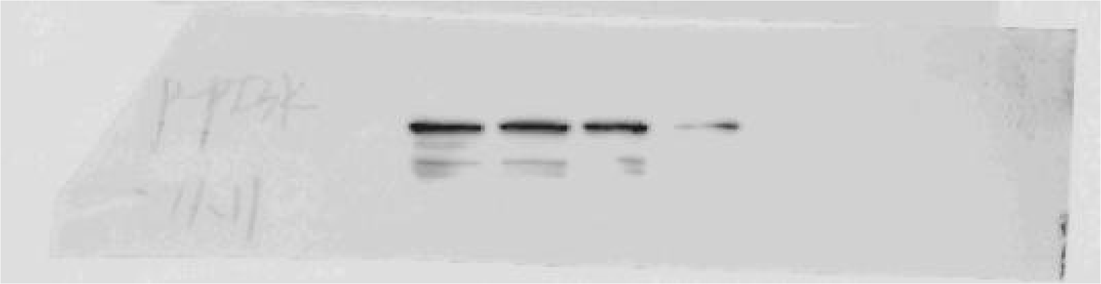

PI3K

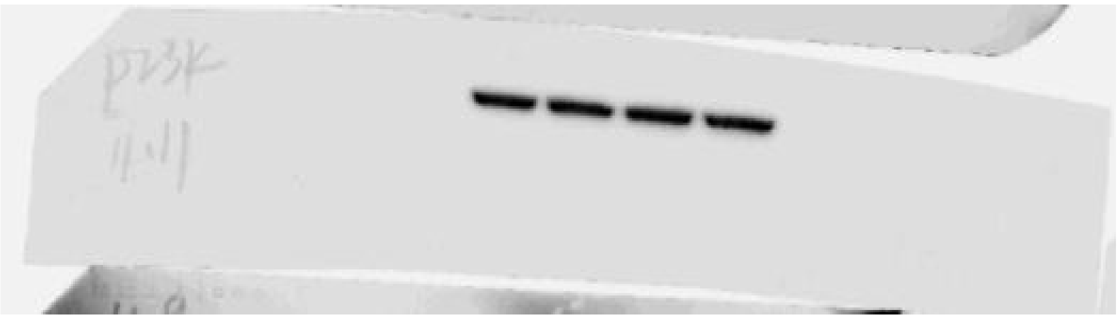

P-AKT

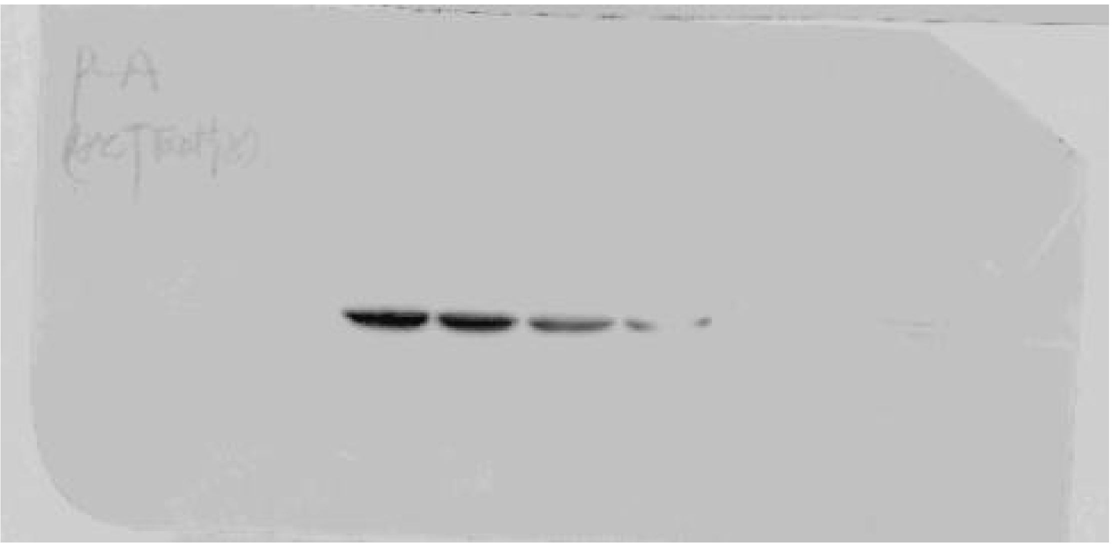

Akt

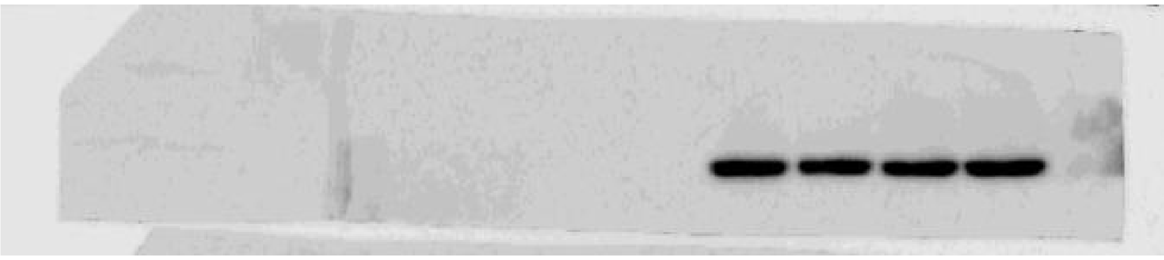

β-actin

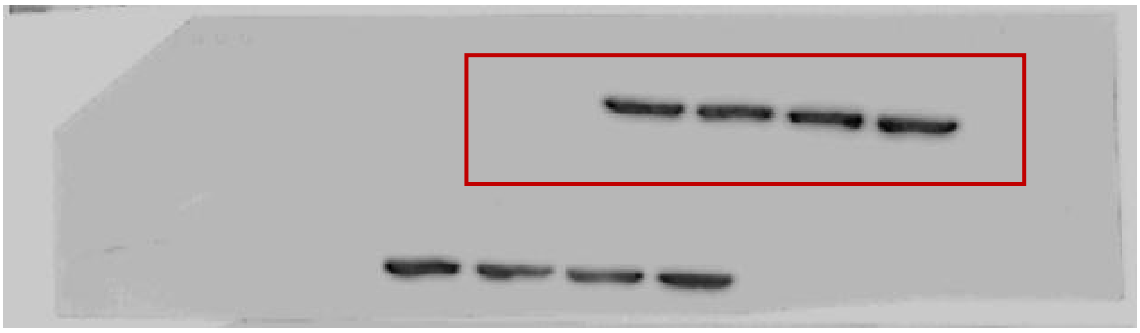

Table S2 Quantitative analysis of relative expression levels of total and phosphorylated EGFR, PI3K and AKT in luteolin-treated A431 cells

Group	p-EGFR/EGFR	p-PI3K/PI3K	p-AKT/AKT
Control	0.912±0.028	0.646±0.016	0.673±0.031
Lu-L	0.759±0.021	0.541±0.010	0.614±0.030
Lu-M	0.556±0.015	0.447±0.014	0.322±0.007
Lu-H	0.300±0.010	0.130±0.003	0.073±0.006
